# A forward genetic screen with a thalamocortical axon reporter mouse yields novel neurodevelopment mutants and a distinct *emx2 *mutant phenotype

**DOI:** 10.1186/1749-8104-6-3

**Published:** 2011-01-07

**Authors:** Noelle D Dwyer, Danielle K Manning, Jennifer L Moran, Raksha Mudbhary, Michael S Fleming, Carlita B Favero, Vita M Vock, Dennis DM O'Leary, Christopher A Walsh, David R Beier

**Affiliations:** 1 Howard Hughes Medical Institute, Department of Neurology, Beth Israel Deaconess Medical Center, Boston, MA, USA; 2 Department of Cell Biology, University of Virginia, Charlottesville, VA 22908-0732, USA; 3 Division of Genetics, Brigham and Women's Hospital, Harvard Medical School, Boston, MA, USA; 4 Molecular Neurobiology Laboratory, Salk Institute, La Jolla, CA, USA; 5 Division of Genetics and Manton Center for Orphan Diseases, Children's Hospital Boston, Harvard Medical School, Boston, MA 02115, USA

## Abstract

**Background:**

The dorsal thalamus acts as a gateway and modulator for information going to and from the cerebral cortex. This activity requires the formation of reciprocal topographic axon connections between thalamus and cortex. The axons grow along a complex multistep pathway, making sharp turns, crossing expression boundaries, and encountering intermediate targets. However, the cellular and molecular components mediating these steps remain poorly understood.

**Results:**

To further elucidate the development of the thalamocortical system, we first created a thalamocortical axon reporter line to use as a genetic tool for sensitive analysis of mutant mouse phenotypes. The TCA-*tau-lacZ *reporter mouse shows specific, robust, and reproducible labeling of thalamocortical axons (TCAs), but not the overlapping corticothalamic axons, during development. Moreover, it readily reveals TCA pathfinding abnormalities in known cortical mutants such as *reeler*. Next, we performed an unbiased screen for genes involved in thalamocortical development using random mutagenesis with the TCA reporter. Six independent mutant lines show aberrant TCA phenotypes at different steps of the pathway. These include ventral misrouting, overfasciculation, stalling at the corticostriatal boundary, and invasion of ectopic cortical cell clusters. An outcross breeding strategy coupled with a genomic panel of single nucleotide polymorphisms facilitated genetic mapping with small numbers of mutant mice. We mapped a ventral misrouting mutant to the *Emx2 *gene, and discovered that some TCAs extend to the olfactory bulbs in this mutant. Mapping data suggest that other lines carry mutations in genes not previously known for roles in thalamocortical development.

**Conclusions:**

These data demonstrate the feasibility of a forward genetic approach to understanding mammalian brain morphogenesis and wiring. A robust axonal reporter enabled sensitive analysis of a specific axon tract inside the mouse brain, identifying mutant phenotypes at multiple steps of the pathway, and revealing a new aspect of the *Emx2 *mutant. The phenotypes highlight vulnerable choice points and latent tendencies of TCAs, and will lead to a refined understanding of the elements and interactions required to form the thalamocortical system.

See Commentary: http://www.biomedcentral.com/1741-7007/9/1

## Background

The conscious perceptions and actions mediated by the cerebral cortex are transmitted and modulated through axonal connections with its intimate processing partner, the dorsal thalamus. These reciprocal projections, the thalamocortical and corticothalamic axons, develop in concert by growing in opposite directions along the same pathway [1]. Some disorders such as epilepsy or schizophrenia may involve defects in the architecture of the thalamocortical system [2-4], but the mechanisms of its development remain poorly understood.

**Figure 1:**
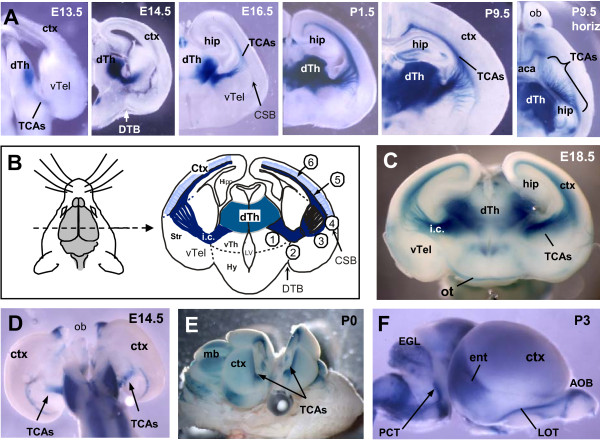
**The TCA-TLZ reporter line marks thalamocortical axons specifically and consistently during development**. **(A) **The TCA-TLZ reporter expresses beta-galactosidase in dorsal thalamic neurons (dTh) starting from E13, and reveals the development of their axon trajectory (TCAs) to cortex (ctx). Cortical axons are not labeled by the reporter. Olfactory axons are labeled in the anterior commissure (aca); some cells in hippocampus (hip) label postnatally. Coronal vibratome sections (100 μm) of brains of indicated ages were stained with X-Gal. The postnatal (P)9.5 sample is cut horizontally to show TCAs fanning out. ob, olfactory bulb. **(B) **Schematic of TCA pathway as viewed in a coronal section of a P0 mouse brain, with developmental steps numbered. See text for details. TCAs 1) grow ventrally; 2) turn to cross the diencephalon-telencephalon border (DTB) by E13.5; 3) defasciculate and fan out in striatum (Str); 4) cross the corticostriatal boundary (CSB) and turn dorsally into cortex; 5) extend dorsally in a restricted layer; 6) make collateral branches into the cortical target area. Hy, hypothalamus; i.c., internal capsule; LV, ventricle. **(C) **The cut face of the caudal half of an E18.5 brain expressing the TCA-TLZ transgene shows the TCA projection from the dorsal thalamus through the ventral telencephalon (vTel) and into cortex. The hippocampus (hip) fills the lateral ventricle. The optic tract (ot) is also labeled by the reporter. **(D) **Dorsal view of a whole-mount E14.5 brain stained with X-Gal reveals the TCAs in the internal capsule (arrows). **(E) **A whole newborn TCA-TLZ brain was cut coronally in half and stained with X-Gal allowing visualization of TCA pathfinding in a whole brain. mb, midbrain. **(F) **A lateral view of a newborn TCA-TLZ brain stained with X-Gal shows labeling in the lateral olfactory tract (LOT) from the accessory olfactory bulb (AOB) and the pontocerebellar tract (PCT). TCAs beneath cortex produce light blue staining. Dark blue staining in entorhinal cortex (ent) is due to cellular staining in a superficial layer; TCAs do not project to entorhinal cortex. EGL, external granular layer of cerebellum.

Humans and mice share a similar organization of the thalamocortical system. The elaborate guidance process of thalamocortical axons (TCAs) in rodents can be broken into six key steps (Figure 1A; detailed reviews in [5-7]). First, the axons extend ventrally along the side of the thalamus. Second, near the hypothalamus, they turn laterally to cross the diencephalon-telencephalon border (DTB) and enter the ventral telencephalon around embryonic day (E)13 [8]. This step appears to require repulsion from Slit in the ventral thalamus [9-11], and interactions with a cluster of 'guidepost cells' in the internal capsule [12-14]. Sema6A is required by a subset of TCAs for the lateral turn, but the mechanism is not understood [15,16]. Third, the large single bundle of TCAs fans out and extends as numerous fascicles through ventral telencephalon (vTel). This extension through vTel may be guided by a permissive 'corridor' of tangentially migrating cells that express the attractant neuregulin-1 [17]. Moreover, the spreading of the TCA array is topographically organized by gradients of cues in the vTel [18-21]. Fourth, the TCA fascicles cross the corticostriatal boundary (CSB) around E15 and turn dorsally. The factors mediating this crossing are unknown, but may be disrupted in mutants for the transcription factors *Tbr1 *or *Fezf2 *[22-25]. Fifth, once in the cortex, the TCAs elongate within the intermediate zone and/or subplate [26-28]. Finally, around birth, TCAs sprout collateral branches within their specific cortical target areas to synapse with layer 4 neurons [27,29]. The TCAs thereby transmit topographic sensory information to the appropriate cortical areas.

Since the understanding of the steps and mechanisms of TCA development remains fragmentary, we performed an unbiased forward genetic screen to identify genes required for thalamocortical development. We combined efficient mutagenesis and mapping strategies [30-32] with a specific axonal reporter, TCA-*tau-lacZ *(TCA-TLZ), to visualize and screen for proper formation of this axon tract inside embryonic mouse brains, without sectioning or immunohistochemistry. Seven independent cortical development phenotypes were found, six with aberrant TCA projections. Initial mapping determined that one mutation was in *Emx2*, while others represent novel genes for this process.

## Results

### The TCA-TLZ reporter line labels thalamocortical axons during development

The TCA-TLZ transgenic reporter line was created fortuitously by pronuclear injection and random genomic insertion of a transgene containing the *golli *promoter driving the *tau-lacZ *reporter gene. This reporter fuses the axonal *tau *microtubule binding protein to beta-galactosidase to localize it to axons [33]. The *golli *promoter is a portion of the myelin basic protein promoter that was shown to promote expression in deep cortical layer neurons [34]. Surprisingly, in this line, the *tau-lacZ *was expressed not in the cortex but instead in dorsal thalamus. The unexpected pattern is presumably due to positional effects of unknown enhancers at the insertion site, mapped to an 8.5-Mb interval of about 45 genes on chromosome 3 (data not shown). The insertion does not appear deleterious: homozygotes are viable and fertile, with no detectable abnormal phenotypes in brain morphology or TCA patterning at birth (n > 40).

The TCA-TLZ reporter line expresses the axonal reporter *tau*-beta-galactosidase in the cell bodies and axons of dorsal thalamic neurons. These neurons are born between E10 and E13 in mice [35]. Expression of the TCA-TLZ transgene is detectable from E13.5 onwards, allowing visualization of TCAs during prenatal development, as they project through vTel and innervate the cortex (Figure 1A). (In this paper, the term 'ventral telencephalon' or vTel refers to the region extending from the ventral surface to the lateral ventricle, including the ganglionic eminences, and the forming basal ganglia and amygdala.) No cortical axons are labeled, although scattered cell bodies in the cortex stain postnatally (Figure 1A, postnatal day (P)9.5). The *tau*-beta-galactosidase labels axons strongly enough to be visible to the naked eye in whole brains (Figure 1C-F). Importantly, the transgene is expressed in the same pattern consistently across different individuals, generations, and genetic backgrounds: in dorsal thalamus, not ventral thalamus (Additional file 1), and in a small number of other neuronal tracts and populations, including the optic tract (Figure 1C, ot), the accessory olfactory bulb and accessory lateral olfactory tract (LOT), the pontocerebellar tract (PCT), and the outer external granular layer of early cerebellum (Figure 1F).

### The TCA-TLZ reporter can reveal the TCA pathfinding and cortical lamination defects found in the *reeler *mutant

**Figure 2:**
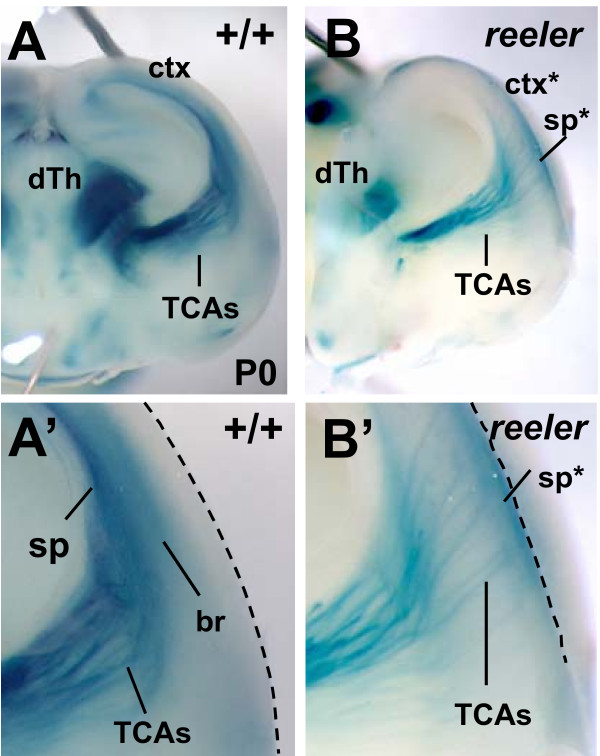
**The TCA-TLZ reporter line reveals the TCA pathfinding and cortical lamination defects of the *reeler *mutant**. **(A, A') **In a P0 control brain, the TCAs elongate (dark blue) in the subplate (sp) layer beneath the cortical plate, and extend collateral branches (br, lighter blue) up to layer 4. **(B, B') **In the *reeler *mutant brain, the cortex is roughly inverted (ctx*), the subplate is abnormally positioned (sp*) at the top of cortex, and the TCA fascicles can be seen crossing the cortical plate to reach it. Dotted lines indicate top of cortical plate at cut edge of hemisphere. dTh, dorsal thalamus.

To test whether the TCA-TLZ reporter can reveal TCA guidance and cortical morphogenesis phenotypes, it was crossed to the well-known cortical lamination mutant *reeler *(Figure 2). In *reeler *mutant brains, cortical layers are roughly inverted, and the subplate cells remain superficial [36]. The TCA-TLZ reporter shows that in control brains at P0 (Figure 2AA'), the TCAs had entered the cortex and could be seen as a dark blue bundle growing in a restricted zone defined by the subplate (Figure 1A', sp), above the mitotic layer and beneath the cortical plate. The collateral branches, thin perpendicular offshoots from the axon shafts, were seen as a lighter blue haze in the deep half of the cortical plate (Figure 1A', br). By contrast, in *reeler *mutant brains, the TCAs did not extend beneath the cortical plate, but grew obliquely across it (Figure 2B, B'), to reach the displaced subplate (sometimes called the superplate, sp*). The appearance of the TCAs in these *reeler *mutants matched that seen with dye tracing previously [28,36]. This experiment demonstrates that the TCA-TLZ reporter can readily reveal both the abnormal TCA pattern and the aberrant cortical layering in the *reeler *mutant, and may be an extremely useful readout of forebrain development abnormalities and tool for analysis of other thalamocortical projection mutants.

### A genetic screen focused on thalamocortical development

**Figure 3:**
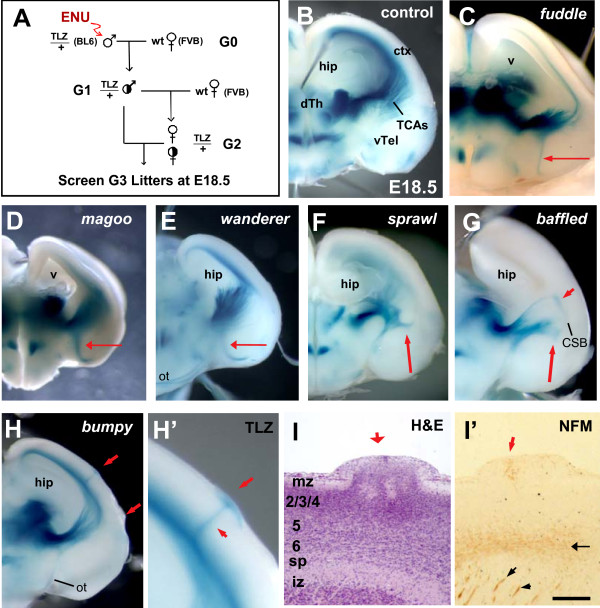
**Mutants found in the thalamocortical screen display a variety of defects in the TCA projection**. **(A) **Intercross breeding scheme for recessive thalamocortical mutant screen. ENU, N-ethyl-N-nitrosourea; wt, wild type. **(B) **A control E18.5 brain stained with X-Gal shows a neatly organized array of TCAs grown from the dorsal thalamus (dTh) through the ventral telencephalon (vTel) into cortex (ctx). The hippocampus (hip) fills the lateral ventricle. **(C) **A *fuddle *mutant brain shows hollow lateral ventricles (v) due to hippocampal hypoplasia, and a partial TCA defect in which a very thin TCA fascicle is misrouted ventrally in vTel (red arrow). **(D) **A *magoo *mutant has a small forebrain with a thick bundle of TCAs misrouted into vTel (red arrow). **(E) **The *wanderer *mutant displays a small cortex and a large bundle of TCAs misrouted ventrally near the DTB (red arrow). **(F) **A *sprawl *brain shows disorganized and overfasciculated TCAs in the lateral vTel, some of which appear stalled (red arrow). **(G) **In a *baffled *mutant brain TCAs are disorganized in lateral vTel (long red arrow), and appear stalled near the corticostriatal boundary (CSB; short red arrow). **(H, H') **In a *bumpy *mutant brain, TCAs project normally to the cortex, but some aberrantly invade the cortical plate to innervate ectopia on the surface of the cortex (red arrows). **(I) **A thin sagittal section through an E18.5 *bumpy *mutant cortical ectopia stained for hematoxylin and eosin (H&E) shows cells erupted through the marginal zone (mz) and pia. iz, intermediate zone; sp, subplate. **(I') **A different section through the same ectopia was stained for axon fibers with neurofilament-M (NFM). Normal fibers can be seen approaching the cortex (black arrowheads), and within the deep cortical layers (black arrow), but fibers are also present in the ectopia (red arrow). Scale bar, 250 μm. (B-H) show one hemisphere of the caudal half of E18.5 G3 brains, cut coronally at the internal capsule. 'ot' indicates optic tract in (E, H), normal in all mutants.

To discover novel genes and phenotypes in thalamocortical development and forebrain morphogenesis, we employed an efficient screening and mapping strategy previously used to identify mouse models of human birth defects [30,32]. First, a three generation breeding strategy of two intercrosses followed by a backcross allowed for efficient collection of recessive mutants and concurrent mapping (Figure 3A). Second, screening was performed on the day before birth so that all of prenatal cortical development could be assayed, but mutations causing postnatal lethality could still be collected. This was important since several mouse knockouts affecting thalamocortical development die at birth. Third, initial mapping was accomplished relatively rapidly through the use of an autosomal genome panel of SNP markers [32]. Finally, incorporating the TCA-TLZ reporter into the scheme enhanced detection and description of prenatal thalamocortical phenotypes.

Males carrying the TCA-TLZ transgene on a C57BL/6 background were mutagenized with N-ethyl-N-nitrosourea (ENU) and mated to wild-type females of the FVB/N strain. G1 males carrying the TCA-TLZ transgene were bred to wild-type FVB/N females, and the resulting G2 daughters were backcrossed to their fathers and sacrificed at embryonic day E18.5 to harvest G3 embryos for screening (Figure 3A and Materials and methods). Embryo brains were cut in half coronally, stained for beta-galactosidase, and examined as whole-mounts. All brains were checked for morphology, and those carrying the transgene (approximately 63%) were examined for abnormalities in the TCA pattern. Five to eight litters from each G1 line were screened. The repeated observation of a specific phenotype in independent litters, followed by faithful transmission after further outcrosses, indicated a high likelihood the abnormality was caused by a monogenic mutation [30].

We screened 57 G1 lines, each representing an independently mutagenized haploid autosomal genome derived from a single G0 sperm. The X chromosome was not assayed in this screen because males were mutagenized and only their male progeny were bred. Seven independent recessive brain development mutants were found, and six of these showed defects in thalamic axons (Table 1 and following sections). Several mutations caused pleiotropic phenotypes, affecting more than one tissue, and three additional mutant lines had only non-brain phenotypes (see Materials and methods). Mutant lines not selected for analysis included a few with exencephaly or embryonic lethal phenotypes. Only those lines that behaved as recessive Mendelian, highly penetrant phenotypes were mapped.

**Table 1:** Mutants found in thalamocortical development screen

Line	Mutant	Forebrain phenotype	TCA phenotype	Other phenotypes	Map interval (Chr: Mb)	Mutant gene
ND15	*magoo*	Smaller overall, enlarged lat. ventricles	Occasional ventral misrouting in vTel	Craniofacial, microphthalmia	19: 32.0-35.9	?
ND21	*outtestine*	Slightly small cortex, OBs	Normal	Short limbs, omphalocoele	12: 101.8-104.5	GMAP210 [37]
ND33	*bumpy*	Cobblestone surface	Fascicles to ectopia outside pia	Stunted tail	Not maintained	?
ND58	*baffled*	Slightly small cortex and OBs, round hippocampus	Disorganized in vTel, some stalled at CSB	Small kidneys, cleft palate, breathing defect	2: 33.8-34.8	?
ND71	*fuddle*	Enlarged lat.ventricles, hippocampal hypoplasia	Ventral misrouting in vTel (partial)	Iris coloboma, cleft palate	19: 20.4-37.3	?
ND87	*wanderer*	Small misshapen cortex and OBs	Severe ventral misrouting near DTB onto ventral surface	Kidney hypoplasia	19: 55-61.3	*Emx2*
ND91	*sprawl*	Normal	Overfasciculation, stalling	Normal	Not mapped	?

All mutants except ND91 were perinatal lethal. Megabase positions based on NCBI build 37; see text for markers. CSB, corticostriatal boundary; DTB, diencephalon-telencephalon boundary; OB, olfactory bulb; vTel, ventral telencephalon.

Since the screen was done as an intercross between the inbred strains C57BL/6 and FVB/N (Figure 3A), genetic mapping by analysis of meiotic chromosomal recombination could be done directly with DNA from affected progeny. By genotyping mutants for a genome-wide panel of up to 768 SNPs that are polymorphic between C57BL/6 and FVB/N, analysis of small numbers of mice resulted in mutation localization to chromosomal intervals of approximately 40 Mb [32]. Microsatellite (simple repeat) markers were then used to confirm and narrow the SNP intervals.

### Mutants display defects at various steps in the TCA projection

The thalamocortical screen revealed several mutant lines with TCA defects visible at low magnification in stained E18.5 brains. TCAs were disrupted at various steps along their pathway (Figure 3B-I). Additionally, several mutants had morphological defects (Table 1), and all were postnatal lethal. The ND21 mutant had normal TCA patterning but a small brain, and is described elsewhere as a mutant in the Golgi protein GMAP210 [37].

Three mutants were found with similar TCA phenotypes at Step 2 of the pathway, in which a subset of TCAs failed to turn laterally upon crossing the DTB (Figure 3C-E). In *fuddle*, *magoo*, and *wanderer *mutants, a single bundle of TCAs was oriented ventrally from the internal capsule just after crossing the DTB, while the remainder of TCAs appeared to navigate normally to the cortex. In the *fuddle *mutant line, the misrouted fascicles were very thin (Figure 3C, arrow), while those observed in *magoo *and *wanderer *appeared thicker with presumably more axons (Figure 3D, arrows). These turning errors could represent failures to detect or respond to ventral repellents, or defects in the interactions with internal capsule guidepost cells. All *fuddle *mutants displayed enlarged lateral ventricles and hippocampal hypoplasia, suggesting other defects in forebrain development. About one-fourth had a TCA defect, and about one-fourth also displayed subtle eye abnormalities, such as irregular irises. The *fuddle *phenotypes co-segregated across generations and mapped to the same region of chromosome 19, indicating that they are all caused by the same mutation.

Steps 3 and 4, in which TCAs spread through vTel and cross the CSB, appeared abnormal in *sprawl *and *baffled *mutants. In both of these mutants, some TCAs were overfasciculated and stalled (Figure 3F). Fewer axons entered the cortex. This phenotype could represent a defect in axon defasciculation from one large bundle to many smaller bundles, or a problem with recognizing the corridor cells or other cues that guide the TCAs through the ventral telencephalon. The *baffled *mutant defect was more dramatic, and seemed most suggestive of a defect in step 4, crossing the CSB (also called the pallial-subpallial boundary). The *baffled *thalamic axons appeared disorganized in the lateral vTel and most failed to enter the cortex (Figure 3G, red arrows).

The *bumpy *mutant phenotype may represent an indirect effect on step 5, the restriction of TCAs beneath the cortical plate. The surface of the *bumpy *mutant forebrain had ectopic lumps of cells outside the pia (Figure 3H-I, red arrows). Cortical lamination appeared disorganized beneath these ectopia. This phenotype is reminiscent of the human brain malformation known as cobblestone (type II) lissencephaly, also called Walker-Warburg syndrome, which is due to cortical neuron over-migration past the marginal zone [38]. Interestingly, a small number of TCAs crossed the cortical plate to invade the 'cobblestones' (Figure 3H, upper red arrow; zoomed in Figure 3H'). Thin sections through cortical ectopia showed they contained both cells erupted through the marginal zone (Figure 3I, red arrow) and axonal fibers (Figure 3I', red arrow). This finding suggests that the misplaced cells may express substrates attractive to TCAs, or that the same mechanism that normally prevents neuron overmigration also acts on TCAs to keep them from invading the cortical plate inappropriately. Axonal innervation of cobblestone-type ectopia has not been shown before in human patients or mouse models, but aberrant cortical wiring could help explain varying seizure phenotypes of some type II lissencephaly patients [39,40].

### *magoo *mutants have small brains and craniofacial defects along with a TCA ventral misrouting defect

**Figure 4:**
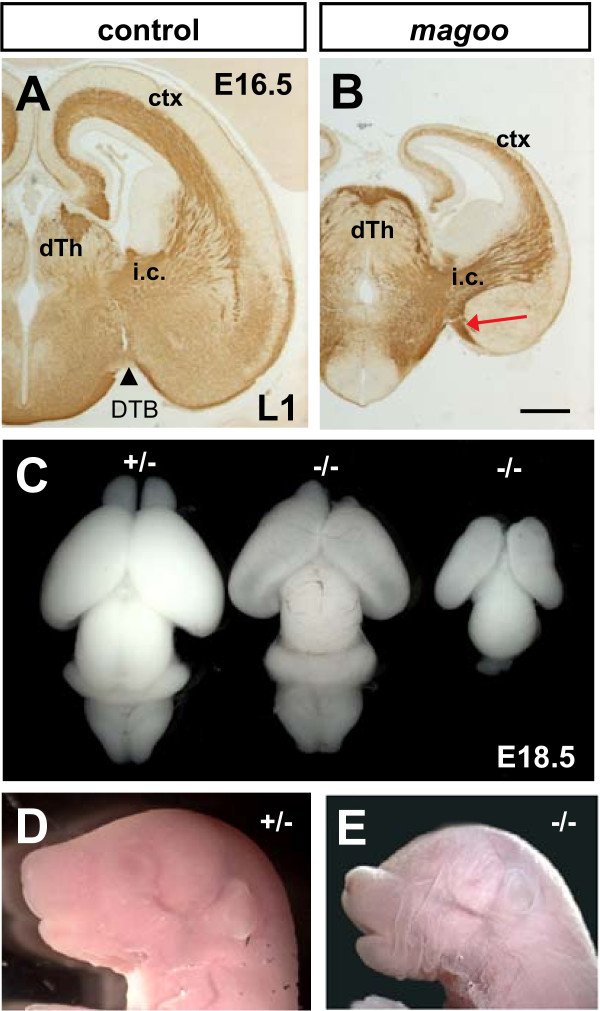
***magoo *mutants have small malformed brains and craniofacial defects**. **(A, B) **L1 immunolabels TCAs and corticothalamic axons in E16.5 brains. The approximate position of the DTB is indicated by a black arrowhead. In the *magoo *mutant brain, an abnormal axon bundle is seen extending ventrally off the internal capsule (i.c.) in the vTel, adjacent to the DTB (red arrow). ctx, cortex. Scale bar, 0.5 mm. **(C) **A heterozygote brain, left, with normal size and morphology was photographed next to two homozygous *magoo *mutant brains from the same E18.5 litter. The homozygote in the center has a slightly smaller brain with hollow lateral ventricles, and its right olfactory bulb is smaller than the left, not damaged. The homozygote brain at right is very small with no olfactory bulbs. **(D) **A normal E18.5 mouse head. **(E) **A homozygous *magoo *mutant E18.5 with small head, shortened snout, and microphthalmia.

*magoo *mutants showed a ventral misrouting defect of TCAs. Out of ten *magoo *mutant embryos stained and expressing the TCA-TLZ reporter, three displayed a small subset of TCAs turned ventrally out of the internal capsule (Figure 3D, arrow). The misrouted bundle appeared to turn ventrally just after the DTB, and then curve slightly rostrally and stop. L1 antibody, which marks several forebrain tracts, including TCAs and corticothalamic axons (Figure 4A), appeared to confirm the ventral misrouting, showing an aberrant thick bundle of axons extending ventrally from the internal capsule along the vTel side of the DTB (Figure 4B, red arrow). The TCAs that did grow to the cortex in the mutant showed no other apparent abnormalities, although the mutant cortex was thinner, with a thinner axonal layer (Figure 4B). Indeed, the entire forebrain was reduced in size in every homozygous *magoo *mutant, but severity varied (Figure 4C). Seventy-one percent (35 of 49) of *magoo *brains were categorized as mild, with only slightly small forebrains (for example, Figure 4C, middle), and the other 29% (14 of 49) were categorized as severe, with hypoplasia of all brain regions (extreme example in Figure 4C, right). Interestingly, the two cortices or olfactory bulbs in a given individual mutant brain were sometimes asymmetric in size (for example, see olfactory bulb asymmetry in Figure 4C, middle). This variability within two halves of one organ suggests that stochastic events underlie the phenotypes. It is not clear whether the TCA defect is cell autonomous or secondary to changes in the forebrain pathway.

In addition to small forebrain size, *magoo *mutants often had craniofacial and eye abnormalities (Figure 4D, E). The snout was usually shortened (76%; 35 of 46), often with cleft palate (24%; 10 of 42). Most mutants had eye phenotypes on one or both sides ranging from iris coloboma to microphthalmia (83%; 38 of 46). Internal organs below the neck appeared normal and proportional to body size, but digits were abnormal in 13% of homozygous mutants (7 of 53). Heterozygotes were indistinguishable from wild types. The phenotypes could indicate a primary defect in patterning, proliferation, or cell migration. However, even in the mildest *magoo *mutants with no craniofacial defects, the forebrain was still slightly small, suggesting that forebrain tissue is the most vulnerable to the loss of this gene.

The *magoo *mutant gene appears to be novel. The mutation was mapped using SNP and microsatellite markers to a small region on chromosome 19 between D19Mit135 and D19Mit12, which does not contain any known thalamocortical development genes. In addition, since the *magoo *map interval overlapped with the larger *fuddle *interval on chromosome 19, and both had a ventral misrouting TCA defect, we tested whether they were allelic by complementation. In five intercross litters, none of 40 embryos showed brain morphology or TCA phenotypes, suggesting that the *magoo *and *fuddle *mutations are in different genes.

### The *baffled *mutant shows severely reduced thalamocortical innervation

**Figure 5:**
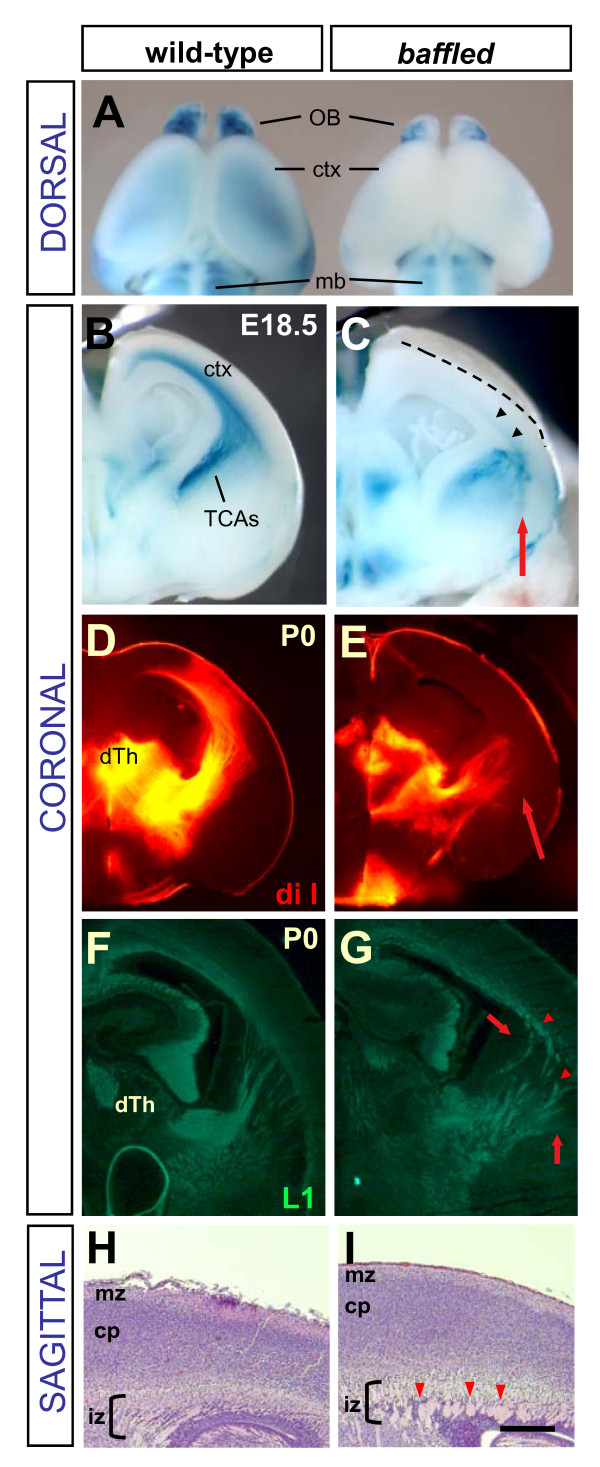
***baffled *mutants have the most severe deficit in thalamocortical innervation**. **(A) **Compared to a wild-type E18.5 forebrain (left), a *baffled *mutant littermate forebrain (right) has a slightly smaller cortex (ctx) and olfactory bulbs (OB), and shows severely reduced beta-galactosidase signal in the cortex, indicating decreased TCA innervation. mb, midbrain. **(B, C) **Coronal views of the cut face of the rostral halves of control and *baffled *forebrains reveal that *baffled *TCAs are disorganized in the lateral vTel, and some appear stalled in thickened bundles near the corticostriatal boundary (red arrow). Only a small number have extended in the cortex (black arrowheads). Dashed line indicates cut edge of brain surface. **(D, E) **Dye tracing with DiI crystal placements in dorsal thalamus (dTh) was done in control and *baffled *mutant fixed P0 brains. Coronal vibratome sections at the level of the internal capsule show that in the *baffled *mutant, dye-labeled axons are disorganized in lateral vTel (E, red arrow), and are not seen in the cortex at this level. **(F, G) **Coronal cryosections of P0 control and *baffled *mutant brains were immunostained for L1-CAM antibody. In mutant sections (G), axons appear disorganized (arrows), and in thicker bundles (arrowheads). **(H, I) **Sagittal thin sections of lateral cortex near the corticostriatal boundary were stained with hematoxylin (purple) and eosin (pink). The axonal layer (intermediate zone, iz) of the control E17.5 cortex (H) contains evenly dispersed thin axon fascicles (pink within bracketed zone), while the *baffled *cortex (I) intermediate zone appears disorganized and contains striking abnormally large axon bundles (red arrowheads). Rostral is to the left. cp, cortical plate; mz, marginal zone. Scale bar, 250 μm.

In contrast to the partial TCA defect seen in *magoo*, the *baffled *mutant showed a fully penetrant and severe TCA phenotype (Figure 3G and 5). As seen from dorsal views of whole brains, wild-type cortices stained blue from innervating TCAs (Figure 5A, left), but *baffled *mutant cortices had greatly reduced cortical staining, suggesting less TCA innervation (Figure 5A, right). While the cortex and olfactory bulbs of *baffled *mutants usually appeared slightly smaller than littermates', they were not misshapen.

Coronal cuts revealed why *baffled *mutants had fewer TCAs in the cortex. Control brains showed a parallel array of TCAs traveling through the lateral vTel, and curving neatly to cross the CSB into the cortex (Figure 5B). By contrast, in *baffled *mutant brains (Figure 5C), the TCAs appeared tangled in the lateral vTel, and some seemed to be stalled in masses near the CSB (Figure 5C, red arrow). Lipophilic dye placements into dorsal thalamus (dTh) to trace TCAs showed a very similar result (Figure 5DE). Similarly, L1 antibody staining (Figure 5FG) confirmed that axon fascicles were disorganized in lateral vTel (5G, arrows), and thickened near the CSB (5G, arrowheads). To examine these axon bundles in cross-section, thin sagittal sections were taken from the lateral cortex of control and mutant brains and stained with hematoxylin and eosin (Figure 5HI). In the lateral cortex near the CSB, the control brain intermediate zone (Figure 5H, bracket) contains evenly dispersed small fascicles, whereas the *baffled *brain intermediate zone contained large swollen bundles of axons (Figure 5I, bracket and red arrowheads). It is uncertain whether these oversized bundles contain only thalamocortical axons, or corticothalamic axons as well.

The thickened disorganized axon fascicles could signify a loss of the ability of TCAs to defasciculate or to interact with the appropriate substrate. The problem appears to arise before the TCAs reach the CSB, but may result in stalling at the CSB. Therefore, the *baffled *mutant may represent a disruption in both step 3, defasciculating and fanning out within the ventral telencephalon, and step 4, crossing the CSB. This unusual TCA phenotype most closely resembles the TCA defects reported in the *Tbr1 *and *Fezf2 *transcription factor knockouts [22-25]. However, *baffled *mapped to an independent locus on chromosome 2, which does not contain a known TCA guidance gene, between D2Mit203 and a marker '58-3' we designed (see Materials and methods).

Aside from the dramatic and very consistent TCA defect, *baffled *mutants had other highly penetrant phenotypes. *baffled *mutants had small kidneys (100%; 27 of 27), hematoma under the nose (95%; 20 of 21), and those collected after birth died within hours (100%; 15 of 15). Heterozygotes appeared normal, indicating a recessive mutation. All phenotypes segregated together and were mapped to the same interval, indicating that they are caused by the same genetic lesion. Candidate genes in the interval are under investigation.

### *wanderer *mutants display TCA fascicles misrouted onto the ventral forebrain surface

**Figure 6:**
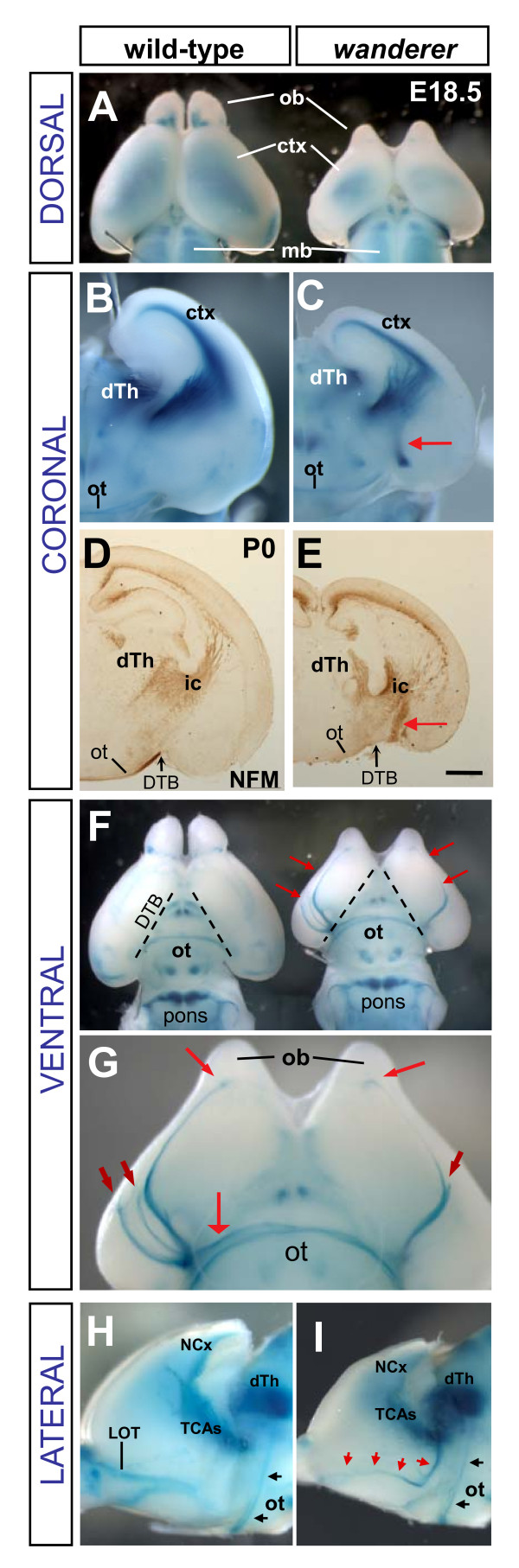
***wanderer *mutants misroute TCA fascicles onto the ventral surface of the forebrain**. **(A) **Dorsal views of a normal littermate (left) and *wanderer *mutant (right) show the *wanderer *mutant has reduced cortex (ctx) and olfactory bulb (ob) size, and reduced blue TCA staining in cortex. mb, midbrain. **(B, C) **All TCAs extend dorsally into the cortex of a wild-type brain, but in a *wanderer *brain, a subset of TCAs turn ventrally (red arrow) after crossing the DTB. dTh, dorsal thalamus; ot, optic tract. **(D, E) **Neurofilament-M (NFM) staining confirms that *wanderer *mutants have a thick bundle of axons extending ventrally from the internal capsule (ic) near the DTB. Scale bar, 0.5 mm. **(F) **Ventral views show aberrant TCA fascicles on the ventral surface of the *wanderer *mutant forebrain (red arrows), but the pontocerebellar projection and the optic tract (ot) appear normal. (The proximal optic nerve was torn off during dissection.) Dashed lines show approximate position of DTB. **(G) **Close-up of *wanderer *brain ventral surface in (F). Some axons grew all the way to the olfactory bulbs (ob) and appeared to make terminal boutons (long red arrows), while others extended shorter distances in a rostral trajectory (short red arrows), and one misrouted TCA fascicle grew along the optic tract (wide red arrow). **(H, I) **The caudal cortex overlying the thalamus was removed to show a lateral view of the pathways of TCAs and optic tract. A control brain (H) shows the TCAs traveling from dorsal thalamus (dTh) to neocortex (NCx), and the optic tract axons (ot, black arrows) coursing up the side of the diencephalon from the optic chiasm to the dLG nucleus of dTh. The lateral olfactory tract (LOT) projects caudally from the olfactory bulb. A similar view of a *wanderer *mutant brain (I) reveals a normal optic tract, but a misrouted bundle of TCAs (red arrows) derailed ventrally from the internal capsule, onto the lateral ventral surface of the forebrain toward the olfactory bulb.

The *wanderer *mutant forebrains had a consistent distinctively abnormal shape. The small oval cortical hemispheres barely touched at the midline, and the olfactory bulbs were short and conical (Figure 6A). The hippocampus was also reduced, but the midbrain and hindbrain appeared normal. Other than a slightly flattened forehead, craniofacial features were normal. The kidneys were small, and any mutants born died within a few hours. The forebrain and kidney phenotypes were fully penetrant (n > 30 mutants), and heterozygotes appeared identical to wild types, indicating a fully penetrant recessive mutation.

In addition to the abnormal forebrain morphology, a striking TCA pathfinding phenotype was observed in *wanderer *mutant brains. A significant reduction of thalamocortical innervation was suggested by reduced beta-galactosidase staining in dorsal cortex (Figure 6A). Coronal views revealed that a subset of TCAs was misrouted ventrally out of the internal capsule (Figure 3E and 6C). Staining for neurofilament-M, which labels many axons, including TCAs and corticothalamic axons, showed a similar aberrant fascicle adjacent to the DTB (Figure 6E, red arrow). However, in contrast to *fuddle *and *magoo *mutants, in which ventrally misrouted axons stalled within ventral telencephalon, the *wanderer *misrouted TCA fascicle grew down onto the ventral forebrain surface and continued in a lengthy rostral trajectory (Figure 6F, G). The fascicle sometimes diverged into multiple bundles (Figure 6F, G, short red arrows). Aberrant ventral surface axons were always observed in both hemispheres in mutants, but never in heterozygotes or wild types (n = 12 -/- and n > 50 +/+ and +/- brains). However, the proportion, number, and precise pathways of the misrouted axons varied. In most mutant hemispheres about half of the axons were misrouted, but one mutant hemisphere had a complete misrouting of all TCAs ventrally with none innervating the cortex (data not shown). Usually the aberrant fascicles grew in a rostro-lateral trajectory; the longest grew all the way to the olfactory bulbs to terminate in bouton structures on the ventral side (Figure 6G, long red arrows). Occasionally a misrouted fascicle stayed in the diencephalon and grew medially along the optic tract (Figure 6G, wide red arrow), but none grew caudally.

The optic tract axons, which are labeled by the TCA-TLZ transgene as well as neurofilament, grow from the optic chiasm up the side of the diencephalon near the point where the *wanderer *misrouted TCAs surface on the medial margin of the ventral forebrain (Figure 6B-I, ot). To ascertain whether the optic tract axons were normal or might contribute to the aberrant fascicles in *wanderer *mutants, the caudal cortex overlying dorsal thalamus was removed to expose the lateral side of the diencephalon (Figure 6HI). The optic tract could be seen coursing up from the optic chiasm on the side of the thalamus to the dorsolateral geniculate nucleus in both control and mutant brains (6 H, I, black arrows). TCAs were seen in both control and mutant exiting dorsal thalamus rostral to the optic tract and then curving toward the neocortex. However, the *wanderer *mutants also showed a TCA fascicle that extended ventrally from the internal capsule and curved rostro-laterally on the ventral surface (Figure 6I, red arrows). A similar pattern was observed in 6 of 6 dissected *wanderer *mutant hemispheres. These data suggest that the optic tract axons are guided normally in *wanderer *mutants and that the aberrant ventral surface fascicles contain only TCAs.

### *wanderer *mutants are homozygous for a nonsense mutation in *Emx2*

**Figure 7:**
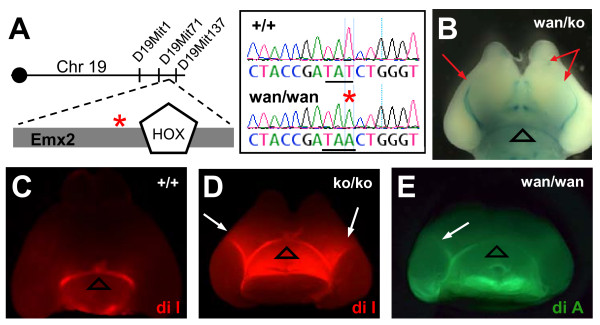
***wanderer *is a nonsense allele of the cortical transcription factor *Emx2***. **(A) **The *wanderer *mutation was mapped to the distal end of chromosome 19 in a region containing the *Emx2 *gene. Sequencing revealed a T-to-A change (red asterisk) in the first coding exon of *Emx2 *in *wanderer *mutants. This mutation (underlined TAT to TAA in chromatogram) is predicted to create an in-frame premature termination codon after 129 residues. **(B) **The *wanderer *allele fails to complement a knockout allele of *Emx2*. A *wan/ko *trans-heterozygote E18.5 brain displayed both the stereotypically shaped small forebrain and the aberrant TCA fascicles (red arrows) on the ventral surface of the forebrain, seen here with the TCA-TLZ transgene. Open arrowhead indicates normal optic tract. **(C-E) **DiI or DiA crystals placed in dorsal thalamus of a wild-type E18.5 brain (C) labeled only the optic tract (open arrowhead at optic chiasm) on the ventral surface of the forebrain. However, in an *Emx2 *homozygous knockout brain (D) or a *wanderer *homozygous mutant brain (E), the dye labels aberrant TCA fascicles growing on the ventral surface (white arrows), as well as the normal optic tract (open arrowhead). Mutant brain in (D) is slightly tilted back relative to brain in (C).

To determine whether *wanderer *represented a known or a new thalamocortical development gene, the mutation was mapped and found on distal chromosome 19 beyond D19Mit1. This 6.3-Mb interval contains the well-known cortical development gene *Emx2 *(Figure 7A, left). Since *Emx2 *knockout mice have a small cortex, small olfactory bulbs, ventral TCA misrouting, kidney dysgenesis, and perinatal lethality, *Emx2 *was a prime candidate gene. Also, the distinctive shape of the *wanderer *mutant forebrain was remarkably similar to that of the *Emx2 *knockout (compare Figure 6A here to Figure 2 in [41]). Sequencing of *Emx2 *from genomic DNA of *wanderer *mutants identified a T to A mutation near the end of the first coding exon (Figure 7A, right). This mutation is predicted to convert residue 130 (Tyr) to a stop codon and result in nonsense-mediated decay, or a protein truncated before the homeodomain and unable to bind DNA. Either way, this allele should act as a null.

To prove that this nonsense mutation in *Emx2 *indeed causes the *wanderer *mutant phenotype, we performed a genetic complementation test between *wanderer *mutants and *Emx2 *knockouts, which have a deletion/insertion in the homeodomain [41]. Heterozygotes for the two alleles were crossed, and progeny were analyzed at day E18.5. Trans-heterozygotes had a visible phenotype indistinguishable from either of the single homozygous mutants, including the distinctively shaped small cortex and olfactory bulbs, and kidney hypoplasia (n = 8 out of 8 trans-heterozygotes). Three of them also carried the TCA-TLZ transgene, which showed long TCA fascicles growing on the ventral forebrain surface (Figure 7B). The failure of the two alleles to complement confirmed that the *wanderer *point mutation in *Emx2 *is causative for the *wanderer *phenotype, and that it behaves as a recessive loss of function mutation.

Surprisingly, the long TCA fascicles on the forebrain ventral surface that appeared striking to us had not been reported in prior studies of the TCA guidance defect in *Emx2 *knockout mutants [42,43]. To ascertain whether this phenotype was present in the homozygous *Emx2 *knockout brains independent of the *wanderer *mutation, we crossed the TCA-TLZ reporter into the *Emx2 *deletion line. Indeed, in brains from homozygous knockout (*ko*/*ko*) animals carrying the reporter, TCA-TLZ fascicles were seen growing in a rostral trajectory on the ventral surface of the forebrain, just as in *wan/wan or wan/ko *brains (n = 8 of 8 *ko*/*ko *hemispheres).

To control for the further possibility that the TCA ventral surface fascicles could be synthetically caused by the *tau-lacZ *transgene, carbocyanine dye tracing was used as an independent method to trace the TCAs. Dye crystal placement in the dorsal thalamus was performed in both *Emx2 *knockout animals that had not been crossed to the TCA-TLZ reporter line, and *wanderer *mutants that did not carry the reporter. In nearly all mutant cases, aberrant dye-labeled fascicles extended rostrally on the ventral surface of the forebrain (Figure 7DE, arrows; 6 out of 6 knockout hemispheres, 5 out of 6 *wanderer *hemispheres). The longest mutant TCAs labeled with DiI were just barely detectable curving toward the olfactory bulb on the whole-mounts. By contrast, heterozygous and wild-type littermate brains (Figure 7C) never exhibited the ventral surface fascicles (for *Emx2 *knockout, 0 of 16 *ko*/*+ *hemispheres, 0 of 12 +/+ hemispheres; for *wanderer*, 0 of 10 +/*wan *hemispheres, 0 of 6 +/+ hemispheres). These findings confirm that loss of *Emx2 *results in some TCAs growing to distant aberrant targets on the ventral forebrain surface, and that *wanderer *has the same TCA phenotype as the *Emx2 *knockout.

## Discussion

We designed a forward genetic strategy to screen directly for mutants with abnormal axon projections inside the mammalian brain. Focusing on the thalamocortical system, we screened with a new reporter line for TCAs. The TCA labeling served not only as a direct indicator of TCA pathfinding abnormalities, but also as an indirect readout of forebrain development defects. Despite the small size of this ENU mutagenesis, independent mutant defects were found at distinct steps of TCA guidance and in forebrain morphogenesis. Mapping indicated that some mutants represent novel genes, and a novel phenotype was caused by mutation of the known thalamocortical development gene *Emx2*. The various phenotypes suggest hypotheses about latent affinities, prerequisites, and the most vulnerable choice points of thalamocortical axons.

### Comparison of the TCA-TLZ reporter to other methods for labeling TCAs

The TCA-TLZ reporter line provides a valuable genetic tool for studying development of TCAs. It has some advantages over other methods for labeling TCAs, such as lipophilic dye tracing, or L1-CAM or neurofilament-M (NFM) antibodies, especially for surveying a large number of embryos. First, this reporter allows visualization of TCAs in whole or half-brains without sectioning or lengthy staining protocols. A second significant feature of this reporter is that it labels virtually all TCAs in every case, with consistent but specific labeling. By contrast, dye tracing is inherently variable in the location and number of cells labeled, and L1 or NFM immunostaining labels many axon tracts. Perhaps most importantly, the TCA-TLZ reporter marks TCAs but not corticothalamic axons, which follow overlapping pathways and cannot be distinguished by immunostaining or dye tracing after age E16.

### Mutants reveal vulnerable choice points during thalamocortical axon navigation

Along their pathway, TCAs have two major turning points between segments of relatively straight growth: at the DTB (step 2) and at the CSB (step 4). These boundaries are defined by abrupt gene expression changes [44]. To cross them, growth cones expand and slow down, perhaps adjusting their affinities for molecular and cellular substrates [8,12,45]. Errors seem to be prevalent at these points: many TCA pathfinding phenotypes from existing knockouts and from this screen cluster near these boundaries.

Making the sharp turn to cross the DTB (step 2) appears to be the step most vulnerable to genetic disruption. While *Mash1 *or *Pax6^smalleye ^*mutants have complete failure of this step [14,46], several other mutants, including *fuddle*, *magoo*, and *wanderer*, have partial ventral misrouting of TCAs at the DTB (Figure 3C-E, 4, 5, and 6). These data suggest that many factors are required for successful navigation at this turning point. One clear requirement for TCAs to cross the DTB is the presence of the internal capsule guidepost cells [14]. One candidate for mediating this interaction is the protocadherin *Celsr3 *[47]. However, the nature of the guidepost cells and the mechanism of their interaction with TCAs remain obscure.

The guidepost cells have also been proposed to facilitate the defasciculation and fanning out of TCAs within ventral telencephalon (step 3) [13]. To defasciculate, the axons may need to switch adhesion preference from one another to the guidepost cells. Alternatively, the spreading of the axon bundle could be mediated by the corridor cells, and/or the gradients of netrin and ephrins in the ventral telencephalon. The relationships and particular roles of these various guidance cells and molecules need to be clarified in future investigations. The TCA overfasciculation, disorganization, and stalling observed in lateral vTel in the *sprawl *and *baffled *mutants (Figure 3F, G) could result from TCAs' failure to interact with any of these intermediate targets, and the future identification of the mutant genes may help to sort out these mechanisms.

We hypothesize that TCA defasciculation in vTel may be a prerequisite for crossing the CSB, since the thicker bundles seen in *sprawl *and especially *baffled *mutants appeared to stall before or near the CSB (Figure 3F, G and 5). Alternatively, the reduced crossing may indicate disruption of a guidance cue there, or the mutant TCAs' ability to detect it. Very little is known about the factors guiding TCA crossing and turning at the CSB. Descending subplate axons [23,48], or ascending lateral ganglionic eminence cell axons [12] have been proposed as substrates, but evidence is indirect, early markers for those cells are lacking, and the question remains as to what guides those pioneer axons. Understanding the defects in mutants such as *baffled *or *sprawl *could help elucidate these mechanisms.

### The TCA-TLZ reporter reveals an undiscovered aspect of the *Emx2 *null phenotype

The *wanderer *mutant was found to carry a nonsense mutation in the well-studied cortical development gene *Emx2*. This finding demonstrates that our screen strategy can identify important thalamocortical development genes, and moreover, that the TCA-TLZ reporter can reveal new details of TCA phenotypes even for well-known genes.

Previous studies of *Emx2 *knockouts did not describe TCA fascicles extended on the ventral surface of the forebrain, despite detailed dye tracing analysis of the TCA phenotype [42,43]. The thin distal segments of the ventral surface TCA fascicles may have escaped detection in thin cross-sections, or they may not have been consistently labeled due to the inherent variability in dye crystals. By contrast, the TCA-TLZ reporter allowed us to examine TCAs in whole-mount brains, and labeled all TCAs consistently (Figure 6).

The secondary behavior of *Emx2 *mutant TCAs we observed after proximal failure to turn laterally at the DTB may reveal latent affinities of the axons. The derailed TCAs were capable of distant extension to incorrect targets. Some stayed in diencephalon and followed the optic tract. Most entered the telencephalon and curved rostrolaterally, some as far as the olfactory bulbs (Figure 6G). Although the olfactory bulbs are not normally connected to thalamus, the wandering TCAs may be attracted to cues for the lateral olfactory tract axons, which normally travel from olfactory bulb to piriform cortex. Indeed, both TCAs and lateral olfactory tract axons respond to slits and netrins [10,49].

The initial turning error of TCAs in *Emx2 *mutants suggests that *Emx2 *is required to ensure that all TCAs turn laterally after crossing the DTB. *Emx2 *is not expressed in the thalamocortical projection neurons [50], so its effect on TCAs must be non-autonomous. The gene is highly expressed in the cortex, but narrow *Emx2 *expression domains have also been found adjacent to the TCA path near the DTB crossing, in the hypothalamus and a narrow strip of medial ventral telencephalon [42,50]. This expression combined with the turning defect suggests that *Emx2 *acts non-autonomously to regulate guidance factors for TCAs at this choice point. A specific role in positioning the internal capsule guidepost cells has been proposed, since they appeared displaced in *Emx2 *knockout brains [42]. Further elucidation of this relationship requires molecular markers for the guidepost cells and discovery of *Emx2*'s transcriptional targets in this ventral telencephalon domain.

### Efficacy of forward genetics combined with a strong axonal reporter

Only about half of the approximately 25,000 protein-encoding genes listed in the Mouse Genome Informatics database have been mutated or even include experimentally based functional annotations [51]. To identify genes and phenotypes related to a specific biological process, the forward genetic approach is complementary to targeted deletions and gene trapping. First, ENU mutagenesis is unbiased with regard to genes since ENU induces point mutations randomly, without hotspots as for gene traps or homologous recombination. Second, several types of alleles are possible with ENU, allowing for partial losses of function and a potentially wider range of phenotypes, which may aid comparisons with human patients. The main drawback of the forward genetic approach, the need for positional cloning, has been rendered straightforward by the mouse genome project, and will be further simplified by the rapidly falling cost of exome and genome sequencing [52,53].

Nevertheless, a successful neurodevelopment screen requires an efficient method to ascertain abnormal phenotypes among thousands of normal specimens. While invertebrate models such as *Caenorhabditis elegans *are transparent, the late-gestation mouse brain is opaque and normally must be sectioned and stained to visualize internal axon tracts. By taking advantage of the TCA-TLZ axonal reporter line first described here, we were able to screen thousands of late-gestation embryos efficiently for axonal phenotypes within the brain. Though many other central nervous system reporter lines [54,55] do not show strong prenatal expression, any that do could also be useful in genetic screens.

Our data suggest a high yield of phenotypes from limited mutagenesis. From 57 lines we found 11 with reproducible late embryonic developmental phenotypes (19%), including 7 affecting the brain (12%). This yield is far higher than in screens for dominant behavioral mouse mutants [56-58], but falls in the range of the few recessive neurodevelopment mouse screens that have been published. For example, mouse screens in younger embryos for interneuron migration defects or peripheral nerve defects identified phenotypes in 3% to 17% of the G1 lines [59-62]. The productivity of a particular mouse screen may reflect the developmental stage examined, the range of phenotypes collected, the sensitivity of the assay, and the number of singly mutable genes required for the process under investigation.

Thalamocortical development is a rich genetic target due to the many steps and cellular interactions required over several days of axon growth through the growing forebrain. Since each G1 line is estimated to carry 30 gene-inactivating mutations [63,64], our screen of 57 lines assayed approximately 1,700 genes, or only 6% of the genome. Thus, many more thalamocortical development and forebrain morphogenesis genes remain to be found through this strategy.

## Conclusions

This screen represents the first attempt at an unbiased assay of the genetic requirements for development of a particular axon tract inside the mammalian brain. Despite assaying only a small fraction of the mouse genome, a variety of phenotypes were found in thalamic axon pathfinding and cortical morphogenesis. The phenotypes represent some novel genes as well as enhanced detection of the known *Emx2 *phenotype. This work paves the way for a more refined understanding of the interactions that TCAs must negotiate on their path through the growing forebrain, and for future genetic screens on other aspects of mammalian brain connectivity and morphogenesis.

## Materials and methods

### TCA-TLZ reporter line construction

A transgene was constructed using the 1.3-kb *golli *promoter [34] fused to the *tau-lacZ *gene [33]. Linearized plasmid was injected into CB6F1 (BALB/c × C57BL/6) egg pronuclei, and eggs implanted into pseudopregnant females (Salk Transgenic Core Facility). Animals carrying the transgene were ascertained by genotyping tail DNA for the presence of the *LacZ *gene. If an animal transmitted the transgene to its progeny, they were examined for expression of the transgene by either X-Gal staining or by RNA *in situ *hybridization for *LacZ *transcript at P0. Out of five transmitting lines, only two showed detectable transgene expression, none in the cortex. One line expressed beta-galactosidase activity in the dorsal thalamus, and was called the 'TCA-TLZ' line and maintained on C57BL/6J.

### Animals and breeding

Embryonic ages were estimated by plug checking (day of plug considered E0.5). Embryos were harvested by caesarean section. Littermate mouse embryos were used as controls for all experiments. Mouse colonies were maintained at the Salk Institute, Brigham and Women's Hospital, and University of Virginia in accordance with National Institutes of Health guidelines and local Institutional Animal Care and Use Committee (IACUC) protocols.

### ENU injections and screening

Heterozygous TCA-TLZ males (n = 39) on a C57BL/6 background were treated with three intraperitoneal injections of either 85 or 90 mg/kg ENU (Sigma, N-3385, Sigma-Aldrich, St. Louis, MO, USA) administered once a week for 3 weeks [65]. Dosages were quantified by spectrophotometry. Of the 39 treated males, 15 survived and recovered fertility between 12 and 16 weeks after the third ENU injection and were bred to wild-type FVB/N females (Taconic, Hudson, NY, USA). G1 males were genotyped for *lacZ *and only carriers selected.

E18.5 G3 embryos were collected, decapitated, and numbered. Tail or skin tissue was saved for DNA isolation. Brains were fixed for 30 minutes in 4% paraformaldehyde in phosphate-buffered saline, cut coronally with a razor blade in the approximate position of the internal capsule, fixed for five more minutes, stained in 0.8 mg/ml X-Gal solution overnight, and examined with bright field stereomicroscopy. Some brains (Figure 1A) were sectioned by vibratome before staining. Results were documented using either a Leica MZ-12 stereomicroscope using a Leica DC500 digital camera, a Leica MZ-16 stereomicroscope with DFC350 digital camera, or an Olympus stereoscope with 35 mm film camera.

### Genetic linkage mapping

Genomic DNA was prepared either by standard proteinase K extraction for microsatellite markers or using the PUREGENE Purification Kit (Gentra Systems, Inc., Minneapolis, MN, USA) for SNP genotyping. SNP genotyping and data analysis were performed as described [32], at the Partners Healthcare Center for Personalized Genomic Medicine (PCPGM) and the Broad Institute Center for Genotyping and Analysis. SNP panels used were a 394 SNP panel (Sequenom, Inc., San Diego, CA, USA) [32], and a 768 SNP panel (Illumina GoldenGate, Illumina, Inc., San Diego, CA, USA). Additionally Line ND71 was genotyped on Illumina LD377 SNP and MD1449 SNP panels in a pilot. dChip software [66,67] was used to visualize the data. Confirmation of SNP results and fine mapping were performed using polymorphic SSLP markers analyzed by PCR on genomic DNA and 3.3% MetaPhor agarose gels.

To define the right end of the *baffled *interval, we designed a new SSLP marker consisting of (GAAA) repeats that we named '58-3' at 34.8 Mb within an intron of the *Hc *gene. Left primer is CCCCTCCGCTTTTCTTTATG; right primer TTGCAAGCATAGCCTCATGT. *Fuddle *was mapped between D19Mit16 and D19Mit88. Map positions for lines ND91(*sprawl*) and ND33(*bumpy*) could not be determined.

Three additional recessive mutants with highly penetrant developmental phenotypes in non-brain tissues were found and mapped. ND3 mutants had stiff dry skin and mapped to chromosome 4: 118 to 143 Mb by SNP analysis. ND24 mutants exhibited cleft palates and mapped between D11Mit30 and D11Mit33. Mutants in line ND94 were anemic and mapped to chromosome 1: 156 to 185 Mb by SNPs.

### Sequencing of *wanderer*

The exons and splice junctions of candidate genes *Emx2 *and *Attractin-like-1 *(*Atrl1*) were sequenced from genomic DNA of mutants and compared to the reference C57BL6 sequence (UCSC Genome Browser [68]). Primers were designed using Primer3 [69]. Exons were amplified by PCR, purified with the Agencourt AMPure kit (Beckman Coulter Genomics, Danvers, MA, USA), and sequenced bidirectionally (SeqWright, Houston, TX, USA). The mutation was confirmed absent in parental strains, homozygous in all mutants and heterozygous in all carriers tested. All trans-heterozygotes from the *Emx2 *complementation test were confirmed by sequencing.

### Dye tracing and histology

For dye tracing of thalamic axons, embryos were fixed by perfusion with 4% paraformaldehyde, brains dissected out, and a razor blade was used to make a coronal cut caudal to the thalamus. Large DiI-C18 crystals (1,1'-dioctadecyl-3,3,3',3'-teramethylindocarbocyanine perchlorate; Molecular Probes, Invitrogen Corporation, Carlsbad, CA, USA) were placed into dorsal thalamus from the caudal side to label most of the dorsal thalamus. Dye was allowed to transport for 2 weeks at 37°C. Brains were imaged whole and phenotypes confirmed by examining vibratome sections. For histochemistry, brains were fixed in 4% paraformaldehyde, and either embedded in paraffin, sectioned at 5 microns, and stained with hematoxylin and eosin (Beth Israel Histology Core), or frozen and cryosectioned at 16 microns for immunostaining. Neurofilament immunohistochemistry (NFM (2H3), 1:100; Developmental Studies Hybridoma Bank) was done on paraffin sections, and L1 immunohistochemistry (Rat anti-L1, 1/5,000; Millipore, Billerica, MA, USA) was done on cryosections, using avidin-biotin reaction (Vector Laboratories, Inc., Burlingame, CA, USA) with DAB substrate as in [14], or an Alexa488 secondary antibody (Figure 5F, G). Photographs were taken with either a Leica MZ12 microscope or a Leica MZ16 fluorescent microscope and Leica DFC300FX digital camera and Photoshop. *In situ *hybridization in Additional file 1 was performed using a radioactive probe for mRNA to the *lacZ *gene, as described in [70]. Each control-mutant pair was photographed at the same magnification.

## Abbreviations

CSB: corticostriatal boundary; DTB: diencephalic-telencephalic border; E: embryonic day; ENU: N-ethyl-N-nitrosourea; NFM: neurofilament-M; P: postnatal day; SNP: single nucleotide polymorphism; TCA: thalamocortical axon; TLZ: tau-lacZ; vTel: ventral telencephalon.

## Competing interests

The authors declare that they have no competing interests.

## Authors' contributions

NDD conceived and coordinated the projects. NDD and DO'L created the TCA-tau-lacZ reporter mouse. DRB and JLM designed the mapping strategy. NDD, DKM, RM, JLM, MSF, CBF, and VMV performed experiments and collected and analyzed data. NDD wrote the paper. DO'L, CAW, and DRB provided guidance and helped edit the manuscript.

## Supplementary Material

Additional file 1 **Supplement to Figure 1: TCA-TLZ transgene is expressed in the dorsal but not ventral thalamus.(A) **A rostral to caudal series of coronal vibratome sections (200 μm) of a P1 TCA-TLZ brain was stained with X-Gal to reveal the thalamic pattern of the TCA-TLZ transgene expressing tau-beta-galactosidase. Expression can be seen in the primary sensory thalamic nuclei, as well as the TCAs. (The blue cellular signal in pretectum (pre) and entorhinal cortex (ent) is not due to TCA innervation or other axons.) **(B) **A similar coronal series of cryosections (20 μm) through the thalamus of a P1 TCA-TLZ brain was probed for *lacZ *mRNA by *in situ *hybridization to allow clearer delineation of thalamic nuclei without axons stained. All of the primary dorsal thalamic nuclei, those that project axons to cortex, express the transgene. Abbreviations: ant, anterior nucleus; dLG, dorsolateral geniculate nucleus; ent, entorhinal cortex; hip, hippocampus; LD, laterodorsal nucleus; LP, lateral posterior nucleus; MG, medial geniculate nucleus; Po, posterior nucleus; pre, pretectum; VL, ventrolateral nucleus; VP, ventroposterior nucleus. Click here for file
