# Supplementary figures and images for: A forward genetic screen with a thalamocortical axon reporter mouse yields novel neurodevelopment mutants and a distinct emx2 mutant phenotype
Source: Neural Dev. 2011 Jan 7;6:3. doi: 10.1186/1749-8104-6-3 (PMC3024922; doi:10.1186/1749-8104-6-3)

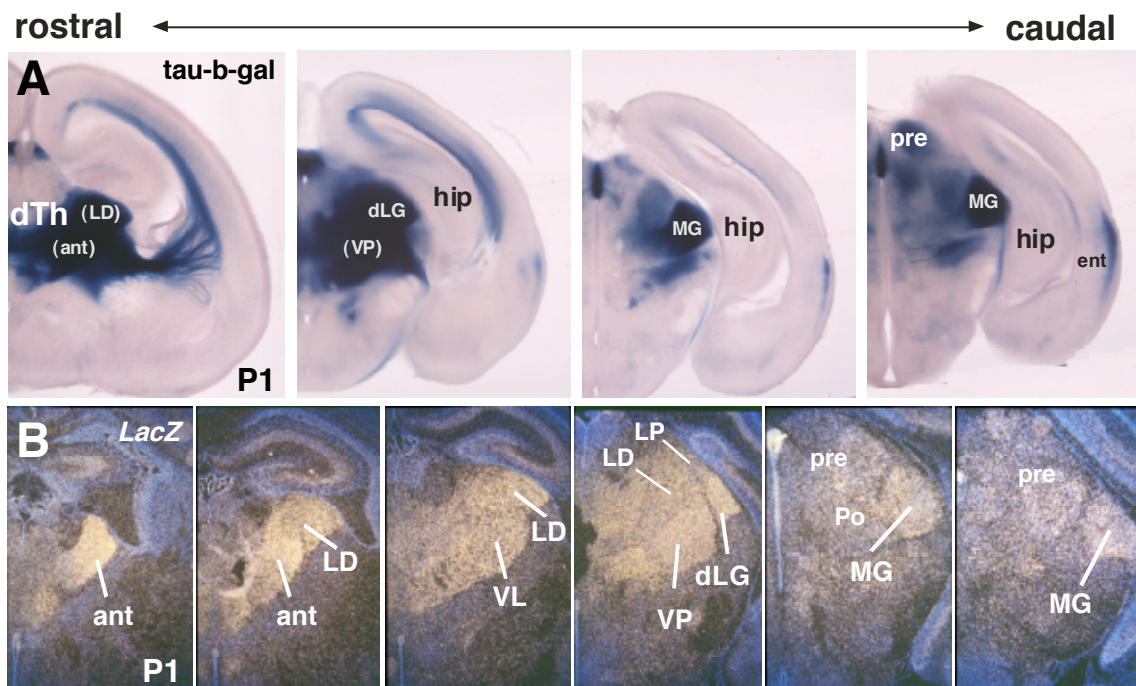

**Additional File 1: Supplement to Figure 1 of Dwyer et al.**

Supplement: Additional file 1 — Supplement to Figure 1: TCA-TLZ transgene is expressed in the dorsal but not ventral thalamus.(A) A rostral to caudal series of coronal vibratome sections (200 μm) of a P1 TCA-TLZ brain was stained with X-Gal to reveal the thalamic pattern of the TCA-TLZ transgene expressing tau-beta-galactosidase. Expression can be seen in the primary sensory thalamic nuclei, as well as the TCAs. (The blue cellular signal in pretectum (pre) and entorhinal cortex (ent) is not due to TCA innervation or other axons.) (B) A similar coronal series of cryosections (20 μm) through the thalamus of a P1 TCA-TLZ brain was probed for lacZ mRNA by in situ hybridization to allow clearer delineation of thalamic nuclei without axons stained. All of the primary dorsal thalamic nuclei, those that project axons to cortex, express the transgene. Abbreviations: ant, anterior nucleus; dLG, dorsolateral geniculate nucleus; ent, entorhinal cortex; hip, hippocampus; LD, laterodorsal nucleus; LP, lateral posterior nucleus; MG, medial geniculate nucleus; Po, posterior nucleus; pre, pretectum; VL, ventrolateral nucleus; VP, ventroposterior nucleus. [file 1749-8104-6-3-S1.PDF]
